# Executive functions and psychopathology: A transdiagnostic network analysis

**DOI:** 10.1371/journal.pone.0338435

**Published:** 2025-12-26

**Authors:** Umer Jon Ganai, Braj Bhushan

**Affiliations:** 1 Department of Psychology, Neuroscience & Behaviour, McMaster University, Hamilton, Canada; 2 Jindal School of Psychology & Counselling, O.P. Jindal Global University, Sonipat, India; 3 Department of Humanities and Social Sciences, Indian Institute of Technology Kanpur, Kanpur, India; FSBSI Scientific Research Institute of Neurosciences and Medicine: FGBNU Naucno-issledovatel'skij institut nejronauk i mediciny, RUSSIAN FEDERATION

## Abstract

Mental health research is shifting toward dimensional, transdiagnostic frameworks, yet the role of executive functions (EFs) across psychopathological domains remains unclear. In this study, we examined transdiagnostic associations and potential directional pathways linking EFs with psychopathology in a large sample of preadolescents (N = 9,119) from the Adolescent Brain Cognitive Development (ABCD) study. We employed a Gaussian graphical model (GGM) to estimate partial correlations and a directed acyclic graph (DAG) to infer potential directional influences between EFs and psychopathology. Modest associations were observed among the EFs and psychopathology. Working memory emerged as a central node, showing positive associations with attention problems, social problems, and rule-breaking behavior, and negative associations with anxious/depressed and somatic complaints. These results were mirrored in the DAG, which identified working memory and attention problems as key converging hubs. Sex-stratified analyses revealed notable differences in network structure. Our findings reveal a core transdiagnostic role for working memory in preadolescent psychopathology.

## Introduction

Preadolescence represents a critical developmental transition marked by significant emotional, behavioral, and neurobiological changes [1–3]. This period is associated with heightened vulnerability to the onset of mental health problems, including internalizing (e.g., anxiety, depression) and externalizing symptoms (e.g., inattention, aggression) [4–6].

In psychopathology research, the predominant diagnostic framework has been shaped by the neo-Kraepelinian tradition, which conceptualizes mental disorders as discrete categories based on the presence or absence of specific symptom clusters [7,8]. This categorical approach, formalized with the publication of the *Diagnostic and Statistical Manual of Mental Disorders* (DSM-III) [9], has guided decades of research into epidemiology, etiology, and treatment of mental disorders. While it has significantly contributed to diagnostic standardization and clinical communication, it has faced increasing criticism for failing to capture the complexity, dimensionality, and heterogeneity inherent in mental health conditions [10,11]. Major concerns include arbitrary diagnostic thresholds, high comorbidity rates, exclusion of subthreshold cases, and questions regarding diagnostic validity and reliability [12,13].

In response, a growing body of research advocates for a dimensional approach to psychopathology. Reflecting this shift, Regier et al. [14, p. 68] noted, “*we are now coming to the end of the neo-Kraepelinian era*,” emphasizing the multifactorial and continuous nature of psychopathological phenomena [15]. Dimensional models conceptualize mental disorders along continua, with symptoms varying in severity rather than as distinct diagnostic categories [16]. These models better reflect empirical evidence showing overlapping symptoms, symptom progression, and developmental variability [17]. Emerging frameworks such as the Research Domain Criteria (RDoC) [17] and the Hierarchical Taxonomy of Psychopathology (HiTOP) [12] aim to move beyond categorical limitations by focusing on transdiagnostic dimensions.

Concurrently with these shifts, preadolescence is a period of rapid development in executive functions (EFs), a set of higher-order cognitive processes that support goal-directed behavior, self-regulation, and adaptive functioning [18–20]. EFs include core components such as inhibitory control (suppressing inappropriate responses), working memory (holding and manipulating information), and cognitive flexibility (shifting between tasks or mental sets) [20, 21]. These capacities are foundational for autonomy and behavioral regulation [22], and their neurobiological underpinnings have been mapped in structural and functional imaging studies [23].

EF impairments have been identified as a shared risk factor across a broad range of DSM-oriented mental disorders, including eating disorders, obsessive-compulsive disorder, depression, anxiety disorders, autism spectrum disorder, and attention-deficit/hyperactivity disorder [24–29]. However, most findings to date have been derived within categorical diagnostic boundaries, which may obscure transdiagnostic patterns. Given this, EF impairments have increasingly been conceptualized as transdiagnostic markers of psychopathology, particularly during preadolescence and adolescence [30,31]. For instance, [32] found that poor EF performance was associated with higher general psychopathology in youth. Similarly, [30] reported negative associations between EF and general psychopathology. However, findings remain mixed. Another study [33] found that externalizing symptoms were linked to poorer inhibition, while internalizing symptoms were related to better performance in inhibition and shifting tasks. In contrast, [34] reported that slower inhibition and shifting predicted internalizing symptoms but did not significantly predict externalizing outcomes in adolescents. More recently, a study [35] observed no significant associations between EF and internalizing, externalizing, or thought disorder spectra.

Given these inconsistencies and reliance on categorical diagnoses in prior research, the current study aims to examine the transdiagnostic nature of EFs in a large sample of preadolescents using network analysis. Network analysis conceptualizes psychological constructs as systems of interacting components, where nodes represent variables and edges reflect associations [36]. First, a Gaussian graphical model (GGM) was estimated, in which regularized partial correlations offer insights into the structure of interrelations while controlling for all other variables [37]. However, GGMs do not provide information about the directionality of associations [38]. To address this, we also used Bayesian network analysis with a directed acyclic graph (DAG), which estimates probabilistic directional relationships between variables and may provide insights into the potential causal pathways [39–41].

Thus, the present study employed both GGM and DAG approaches to investigate the complex, transdiagnostic associations between EFs and dimensional psychopathology in a large sample of preadolescents. Specifically, we aimed to: (1) identify the network structure of EFs and psychopathology using partial correlation-based GGM, (2) estimate node centrality to determine which EF components or psychopathology domains occupy the most influential positions in the network, (3) apply DAG analysis to explore potential directional or causal pathways among variables, particularly focusing on whether specific EFs may act as upstream cognitive mechanisms contributing to various forms of psychopathology, and (4) conduct sex-stratified network analyses to examine potential differences in network structure between males and females. This comprehensive approach offers a more nuanced understanding of how EFs relate to the dimensions of psychopathology during a critical developmental period.

## Methods

### Participants and procedures

The Adolescent Brain Cognitive Development (ABCD) study is a longitudinal, multisite project in the United States that collects clinical, behavioral, neuroimaging, and genetic data from children aged 9–10 years [42,43]. The study includes over 11,000 participants from diverse demographic backgrounds, recruited through a school-based selection process designed to ensure representativeness and reduce selection biases [43].

Data for the present study were obtained from the ABCD study’s “Curated Annual Release 4.0” (https://nda.nih.gov/study.html?id=1299) [42,43]. Baseline assessments were used in the present study. Participants with neurological conditions (e.g., cerebral palsy, epilepsy, multiple sclerosis), traumatic brain injury, or incomplete data were excluded, resulting in a final sample of 9,119 participants.

Parents or guardians provided written informed consent after the study procedures were fully explained, and participants gave their assent before participating. All procedures for the ABCD study were approved by the central institutional review board at the University of California, San Diego (IRB# 160091), and by the institutional review boards of each of the ABCD study sites [42,43]. The current study used publicly available, de-identified data and did not involve direct interaction with human participants. Therefore, no additional ethical approval was required for this analysis. The de-identified data used in the present study are available through the National Institute of Mental Health repository (https://nda.nih.gov/) after obtaining approval to access the ABCD study data.

### Measures

#### Executive functions.

Executive functions (EFs) were assessed using the National Institutes of Health (NIH) Toolbox (https://nihtoolbox.desk.com), a standardized set of tasks designed to measure various cognitive processes [44–46]. Detailed descriptions of this neurocognitive battery within the ABCD study are available elsewhere [47]. The tasks utilized in the present study included the NIH Toolbox Flanker Task**,** NIH Toolbox List Sorting Working Memory Test**,** NIH Toolbox Dimensional Change Card Sort Task**,** NIH Toolbox Pattern Comparison Processing Speed Test**,** and NIH Toolbox Picture Sequence Memory Test**.** These tasks are described below.

*Inhibitory control* was evaluated using the NIH Toolbox Flanker Task, a modified version of the Eriksen Flanker Task, which measures the degree to which participants’ responses are influenced by the congruence or incongruence of flanking stimuli relative to a central target [48]. In each trial, four flanking stimuli (two arrows on the outer left and two on the outer right) either align with the direction of the central target arrow (congruent trial) or point in the opposite direction (incongruent trial). Participants were instructed to press a key corresponding to the direction of the central arrow. The ABCD study employed a composite score for the Flanker Task, integrating response speed and accuracy. Age-corrected standardized scores were used in the present study, and higher scores indicate better performance. The task demonstrates strong test-retest reliability in adolescent samples (ICC = 0.92) [49].

*Working memory* was assessed with the NIH Toolbox List Sorting Working Memory Test, an adaptation of the letter-number sequencing test that uses pictorial stimuli instead of words or letters [50–52]. This task requires participants to sequence stimuli by size within specific categories. Participants viewed images of animals and foods of varying sizes on an iPad and were asked to list them verbally in order from smallest to largest. Trials began with a single category (e.g., animals) and two items; if the participant responded correctly, the number of items increased incrementally to seven. The process was repeated in a second condition, where participants sequenced items from two categories (e.g., animals and foods) in order from smallest to largest, listing all items from one category followed by the other. The ABCD study provided a total score based on the sum of correct responses across both list sorting tasks. Age-corrected standardized scores were used in the present study, and higher scores indicate better performance. The task demonstrates good test-retest reliability in the adolescent population (ICC = 0.86) [49].

*Cognitive flexibility* was assessed using the NIH Toolbox Dimensional Change Card Sort Task (DCCS) [53]. In this task, participants were shown two objects at the bottom of a screen (e.g., a white rabbit and a green boat) and asked to sort a third object, displayed in the center, by matching it to one of the bottom objects based on either color or shape [49,54]. The task consisted of three blocks of trials- participants first sorted objects by one dimension (e.g., color), then by another (e.g., shape), and finally by dimensions in random order. The ABCD study provided a composite score for the DCCS, integrating both response speed and accuracy. Age-corrected standardized scores were used in the present study, and higher scores indicate better performance. The task demonstrates strong test-retest reliability in adolescents (ICC = 0.92) [49].

*Processing speed* was assessed using the NIH Toolbox Pattern Comparison Processing Speed Test, an adaptation of the Pattern Comparison Task originally developed by Salthouse [55–58]. Participants were presented with two pictures and asked to indicate by touch input whether the pictures were identical or different. The score reflects the number of items correctly completed within a fixed time limit. Age-corrected standardized scores were used in the present study, and higher scores indicate better performance. The task demonstrates good test-retest reliability in children and adolescents (ICC = 0.84) [55].

*Episodic memory* was evaluated using the NIH Toolbox Picture Sequence Memory Test. In this task, participants viewed a series of 15 images depicting activities or events within a specific context (e.g., working on a farm) and were required to reproduce the sequence in the order presented [59,60]. The ABCD study provided a composite score reflecting the accuracy of sequence reproduction, adjusted for the number of correctly ordered pairs. Age-corrected standardized scores were used in the present study, and higher scores indicate better performance. The task demonstrates adequate test-retest reliability in pediatric populations (ICC = 0.76) [59].

### Psychopathology.

The Child Behavior Checklist (CBCL; ages 6–18 form) was used in the ABCD study to assess a wide range of emotional and behavioral symptoms in participants [61]. The CBCL includes 119 items that parents rated as 0 (“not true”), 1 (“somewhat or sometimes true”), or 2 (“very true or often true”), based on the extent to which various behaviors (e.g., “argues a lot”) characterized their child over the preceding six months. The CBCL comprises eight validated syndrome scales: *Anxious/Depressed, Withdrawn/Depressed, Somatic Complaints, Social Problems, Thought Problems, Attention Problems, Rule-Breaking Behavior, and Aggressive Behavior* [62]. For each syndrome scale, summed raw scores were reversed so that higher scores indicated fewer problems. For example, in the *Anxious/Depressed* scale, the raw score was subtracted from the maximum possible score (26) to produce a reversed score, where higher values reflect fewer anxious or depressive symptoms. These reversed summed scores were used in the present study. This reversal was implemented to facilitate interpretation in our analyses. The CBCL syndrome scales demonstrate good psychometric properties and high internal consistency in the ABCD study sample (α = 0.88) [62,63]. The study measures and their abbreviated ABCD names are provided in the Supporting Information.

### Statistical analyses

Data analyses were conducted using the open-source statistical software *R* [64]. First, the normality of the eight CBCL syndrome scales was assessed. Normality assumptions were not met, as skewness exceeded 2 and/or kurtosis exceeded 7, based on established benchmarks [65]. Consequently, following the guidelines for psychological network analysis [66], a nonparanormal transformation was applied to all CBCL syndrome variables using the *R* package *huge* [67]. Next, z-score standardization was performed on EF scores. A power analysis was conducted using the *powerly* package in *R* [68] to determine the sample size required for a GGM. This procedure involved three steps. First, Monte Carlo simulations were conducted across various sample sizes to evaluate the model’s sensitivity (ability to detect true edges) and statistical power (probability of achieving this performance level). Second, a monotonic spline smoothing technique was applied to the simulation results to identify the smallest sample size meeting the predefined sensitivity and power thresholds. Third, additional simulations validated the selected sample size to confirm the consistency and robustness of the estimated performance [62].

Next, a GGM was estimated to examine the relationships among the EFs and eight CBCL syndrome scales. In a GGM, each variable is represented as a node, and edges between nodes indicate conditional associations, i.e., associations that remain after controlling for all other variables in the network [69]. The model was estimated using a regularized approach, specifically the graphical Least Absolute Shrinkage and Selection Operator (LASSO) [70], combined with the Extended Bayesian Information Criterion (EBIC) [71]. This regularization procedure shrinks small partial correlations to zero, thereby retaining only the most robust and meaningful edges [72]. Network estimation and visualization were performed using the *qgraph* and *bootnet* packages in *R* with default settings [69,70]. In the resulting network plot, positive associations are depicted with blue edges, and negative associations with red edges. The thickness of an edge reflects the strength of the partial correlation between the corresponding nodes.

The centrality of the network was assessed using bridge strength and one-step bridge expected influence (BEI). Bridge strength quantifies a node’s total connectivity to another cluster by summing the absolute edge weights of its cross-cluster links. BEI evaluates a node’s direct influence by summing signed edge weights, capturing positive and negative associations [73]. High bridge strength indicates strong cross-cluster connectivity, while high BEI highlights nodes with significant positive or negative influence on other clusters. These bridge centrality metrics were estimated using the *networktools* package in *R* [73].

The stability of the networks was measured using correlation stability coefficients (CS), while the accuracy of edge weights and centrality was assessed through bootstrapping. For both edge weights and centrality, 2,500 bootstrap iterations were performed at an *α* level of 0.05 with a 95% confidence interval. Subsequently, a node-dropping subset bootstrap method was applied to assess centrality stability and compute the CS coefficient for the networks. A CS coefficient above 0.25 is deemed acceptable, with values preferably exceeding 0.5 [72].

To assess potential causal relationships, a DAG was estimated to model the probabilistic dependencies among network nodes [74]. The DAG was constructed using the *hill-climbing algorithm* implemented in the *R* package *bnlearn* [75]. This algorithm employs a bootstrap function that optimizes the network structure by iteratively adding, removing, or reversing edges to maximize the Bayesian Information Criterion (BIC) as the goodness-of-fit score. The bootstrap process involves 50 random restarts, each with 100 perturbations, to avoid local maxima and explore diverse node connections [38,41,76,77]. Through these iterations, the algorithm identifies the best-fitting network structure with the optimal BIC value [41].

To ensure the stability of the resulting DAG, the guidelines for implementing DAGs in psychological research were followed, and 10,000 bootstrapped samples with replacement were computed to create a network for each sample, which were then averaged to produce a final network structure [41]. This involves a two-step approach. First, the frequency of each edge’s appearance in the 10,000 bootstrapped networks was ascertained. The optimal cutoff method of [78] for retaining edges was used, yielding both high sensitivity and specificity. Second, the direction of each surviving edge in the 10,000 bootstrapped networks was determined, an edge from node X to node Y was considered if it appeared in at least 51% of the bootstrapped networks [79].

The averaged DAG networks were visualized in two formats. In the first visualization, edge thickness reflects relative Bayesian Information Criterion (BIC) values, where thicker edges indicate greater importance, as their removal would significantly impair model fit [38,79]. In the second visualization, edge thickness represents directional probabilities, with thicker edges signifying a higher likelihood of a specific direction (e.g., from node X to node Y) [79]. Since the data are cross-sectional in nature, the directions should be interpreted as probabilistic dependencies rather than causal effects. When two nodes show comparable support for both possible directions, bootstrap averaging may yield weak or null directional probabilities. Such instances can be interpreted as ambiguous or undirected associations rather than as evidence of no relationship.

Lastly, separate GGMs were estimated for male and female samples using the same procedure. Differences between the male and female networks were evaluated using the Network Comparison Test (NCT) [80].

## Results

### Sample characteristics

**Fig 1:**
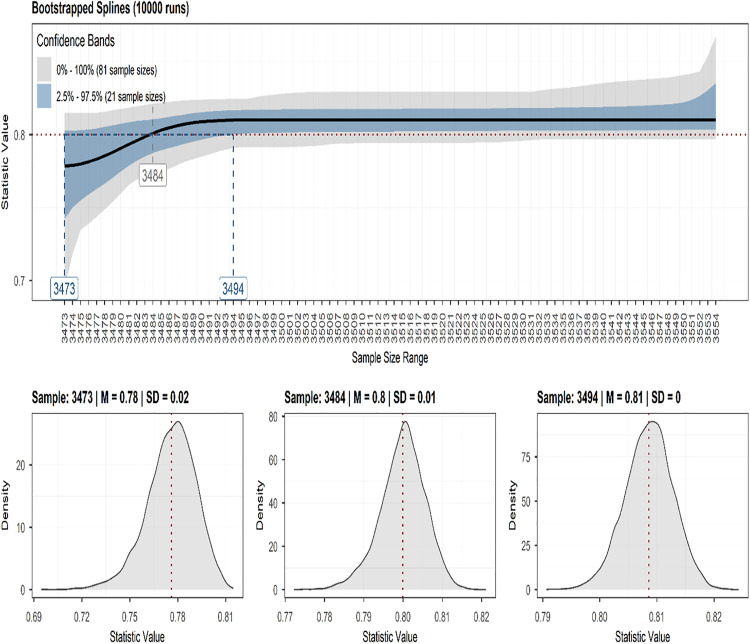
Statistical power analysis and sample size determination. The statistic value represents network sensitivity, defined as the proportion of edges in the true network structure correctly identified as non-zero. The red dotted line denotes the target sensitivity level.

Table 1 summarizes the demographic characteristics of the participants. The average age of the participants was 9.92 years, with the majority being male and White. Based on our pre-specified sensitivity level of 0.7 and desired statistical power of 0.8, a minimum sample size of 3,484 participants was recommended (Fig 1). Our final sample size surpassed this threshold, ensuring sufficient statistical power.

**Table 1:** Demographics characteristics of the participants.

*Demographics*	*N or M (SD) or %*
N	9,119
Age (Years)	9.92 (0.62)
Sex (Male/Female)	4,754/4,365
*Household Income*	
≤ $50,000	2,298 (25.20)
≥ $50,000 to <$100,000	2,381 (26.11)
≥ $100,000	3,680 (40.36)
Do not know and refuse to answer	760 (8.33)
*Race/Ethnicity*	
White	4,913 (53.88)
Black	1,233 (13.52)
Hispanic	1,842 (20.20)
Asian	207 (2.27)
Other	924 (10.13)

### Regularized partial correlation network

**Fig 2:**
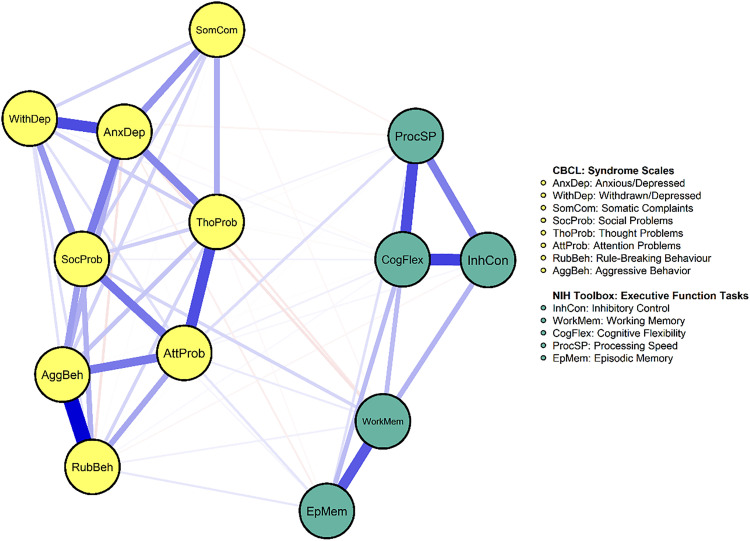
Regularized partial correlation network of EFs and CBCL syndrome scales, with nodes representing EFs and psychopathology dimensions. Blue edges indicate positive associations and red edges negative associations.

The regularized partial correlation network of EFs and CBCL syndrome scales is visualized in Fig 2, with the corresponding partial correlation matrix provided in S1 Table. The network visually revealed two distinct clusters, EFs and CBCL syndrome with stronger connections within clusters than between them. Several cross-cluster associations were noteworthy. *Working memory* exhibited six non-zero edges with CBCL syndrome scales, including a strong positive association with *Social Problems* (0.06) and two negative associations with *Anxious/Depressed* (−0.04) and *Thought Problems* (−0.03). No connections were observed between *working memory* and *Withdrawn/Depressed* or *Aggressive Behavior*. *Inhibitory control* displayed three non-zero edges, with positive associations with *Attention Problems* (0.02) and *Social Problems* (0.02) and a negative association with *Rule-Breaking Behavior* (−0.02). *Cognitive flexibility* had the highest number of non-zero edges (seven), including three negative associations with *Anxious/Depressed* (−0.01), *Thought Problems* (−0.01), and *Aggressive Behavior* (−0.01). However, no connection was found with *Somatic Complaints*. *Processing speed* showed six non-zero edges, with notable positive associations with *Attention Problems* (0.05) and *Social Problems* (0.02). Lastly, *episodic memory* exhibited multiple positive and negative edges with CBCL syndrome scales.

Within-cluster connections were stronger, as expected. For example, among the EFs, *inhibitory control* and *processing speed* showed a robust positive partial correlation (0.30), while among the CBCL syndrome scales, *Aggressive Behavior* and *Rule-Breaking Behavior* had the strongest association (0.40). These findings highlight the relative independence of EFs and CBCL syndrome scales, with limited but meaningful cross-cluster interactions, particularly involving *working memory*, *inhibitory control*, and *cognitive flexibility*.

**Fig 3:**
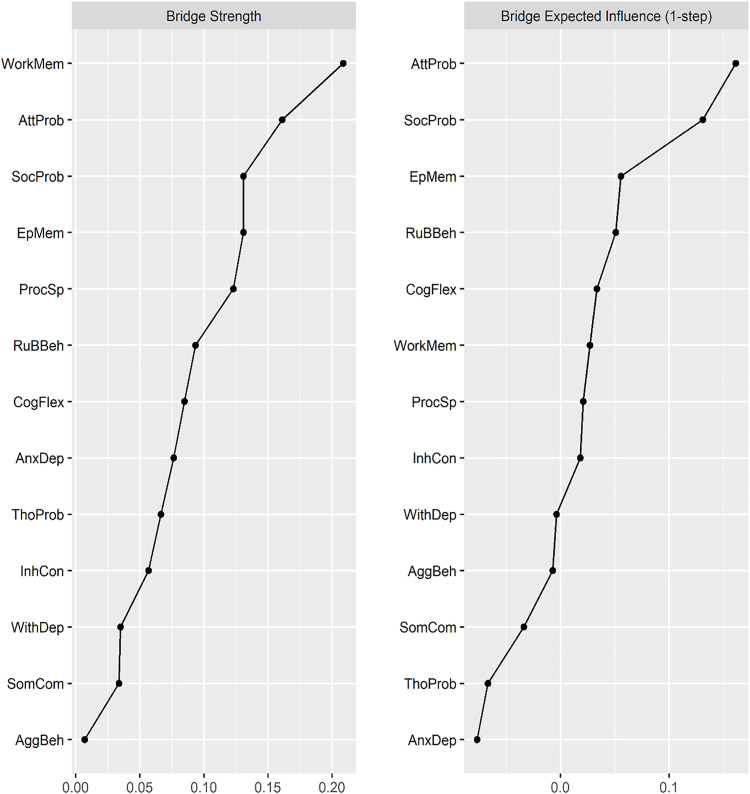
Centrality measures of bridge strength and bridge expected influence (BEI) for the EF-psychopathology network. AnxDep: Anxious/Depressed; WithDep: Withdrawn/Depressed; SomComp: Somatic Complaints; SocProb: Social Problems; ThouProb: Thought Problems; AttProb: Attention Problems; RuBBeh: Rule-Breaking Behavior; AggBeh: Aggressive Behavior; InhCon: Inhibitory Control; WorkMem: Working Memory; CogFlex: Cognitive Flexibility; ProcSp: Processing Speed; and EpMem: Episodic Memory.

Fig 3 displays the bridge strength and BEI of the EF and CBCL syndrome scales network. *Working memory* exhibited the highest bridge strength (0.21), followed by *Attention Problems* (0.16) and *Social Problems* (0.13), indicating these nodes have the strongest overall connections. Conversely, *Aggressive Behavior* showed the lowest bridge strength (0.01). For BEI, *Attention Problems* had the highest value (0.16), followed by *Social Problems* (0.13) and *episodic memory* (0.06), while *Anxious/Depressed* had the lowest BEI (−0.08).

**Fig 4:**
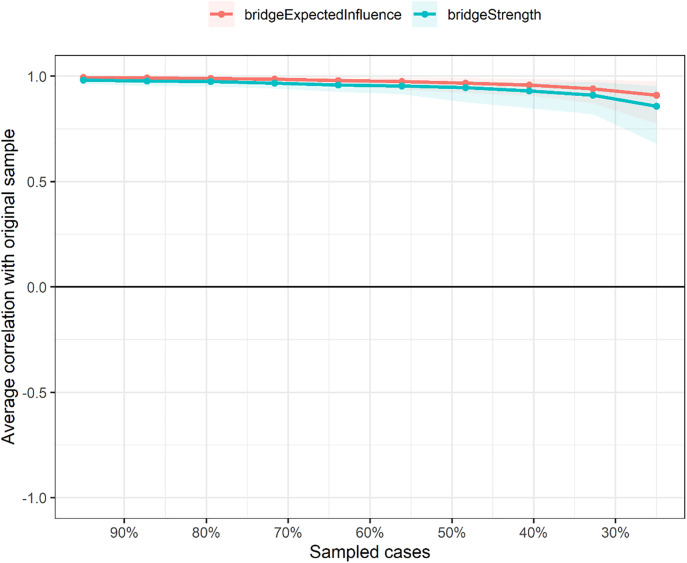
Bootstrapped stability plot of bridge strength and bridge expected influence (BEI) for the EF-psychopathology network.

The correlation stability (CS) coefficients for edge weights, bridge strength, and BEI were 0.75 (CS [cor = 0.7]), indicating that the network is highly stable. This suggests that 75% of the sample could be dropped while maintaining a correlation of at least 0.7 with the original network estimates, reflecting robust results. Bootstrapped stability plots for edge weights are provided in S1 Fig, and those for bridge strength and BEI are provided in Fig 4.

### Bayesian network

**Fig 5:**
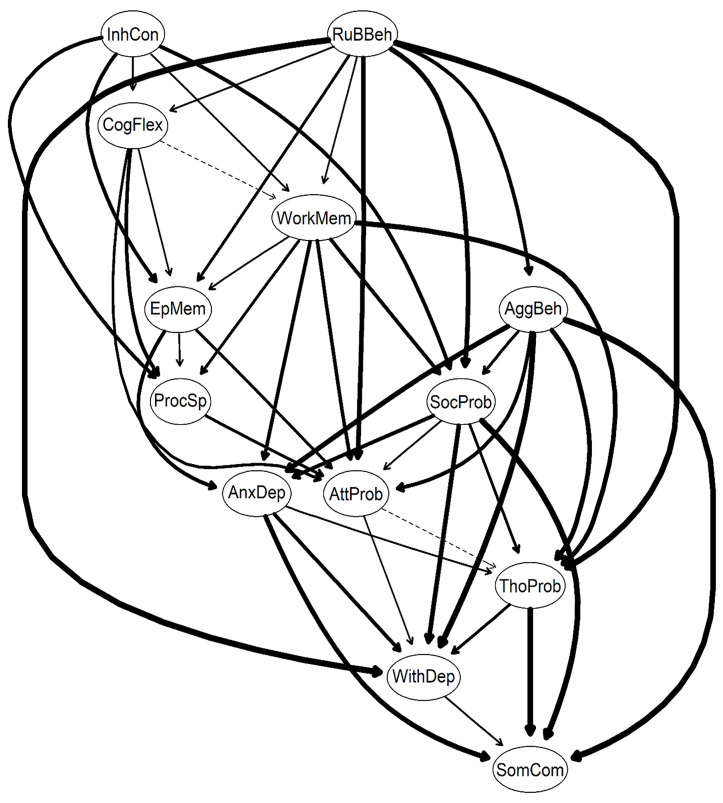
Directed Acyclic Graph of EFs and CBCL syndrome scales with arrow thickness indicating directional probability. AnxDep: Anxious/Depressed; WithDep: Withdrawn/Depressed; SomComp: Somatic Complaints; SocProb: Social Problems; ThouProb: Thought Problems; AttProb: Attention Problems; RuBBeh: Rule-Breaking Behavior; AggBeh: Aggressive Behavior; InhCon: Inhibitory Control; WorkMem: Working Memory; CogFlex: Cognitive Flexibility; ProcSp: Processing Speed; and EpMem: Episodic Memory.

Fig 5 illustrates the directional probabilities of edge weights in the DAG, derived from averaging 10,000 bootstrapped networks, with higher values indicating a greater likelihood that the edge points in the direction specified in Table 2. The thickest edge, representing the strongest directional probability, extends from *Aggressive Behavior* to *Somatic Complaints* (directional probability = 0.995), followed by the edge from *Rule-Breaking Behavior* to *Withdrawn/Depressed* (directional probability = 0.99). Other notable edges between EFs and CBCL syndrome scales include those from *working memory* to *Anxious/Depressed* (directional probability = 0.90) and from *working memory* to *Social Problems* (directional probability = 0.79). Additionally, both *processing speed* and *episodic memory* exhibit edges pointing to *Attention Problems*, with directional probabilities of 0.70 and 0.76, respectively.

**Table 2:** Directional probabilities of arrows in the DAG.

Arrows in the DAG		Values determining arrow thickness
From	To	Directional Probability
AnxDep	WithDep	0.83
AnxDep	SomCom	0.92
AnxDep	ThoProb	0.63
WithDep	SomCom	0.63
SocProb	AnxDep	0.79
SocProb	WithDep	0.93
SocProb	SomCom	0.96
SocProb	ThoProb	0.75
SocProb	AttProb	0.57
ThoProb	WithDep	0.75
ThoProb	SomCom	0.98
AttProb	WithDep	0.66
AttProb	ThoProb	0.51
RuBBeh	WithDep	0.99
RuBBeh	SocProb	0.91
RuBBeh	ThoProb	0.97
RuBBeh	AttProb	0.94
RuBBeh	AggBeh	0.84
RuBBeh	WorkMem	0.66
RuBBeh	EpMem	0.75
AggBeh	AnxDep	0.92
AggBeh	SomCom	0.995
AggBeh	SocProb	0.75
AggBeh	ThoProb	0.91
AggBeh	AttProb	0.86
InhCon	WorkMem	0.65
InhCon	CogFlex	0.62
InhCon	ProcSp	0.82
WorkMem	AnxDep	0.90
WorkMem	SocProb	0.79
WorkMem	EpMem	0.65
CogFlex	WorkMem	0.52
CogFlex	ProcSp	0.77
CogFlex	EpMem	0.65
ProcSp	AttProb	0.70
EpMem	AttProb	0.76
EpMem	ProcSp	0.66

***Notes:*** AnxDep: Anxious/Depressed; WithDep: Withdrawn/Depressed; SomComp: Somatic Complaints; SocProb: Social Problems; ThouProb: Thought Problems; AttProb: Attention Problems; RuBBeh: Rule-Breaking Behavior; AggBeh: Aggressive Behavior; InhCon: Inhibitory Control; WorkMem: Working Memory; CogFlex: Cognitive Flexibility; ProcSp: Processing Speed; and EpMem: Episodic Memory.

Two distinct clusters emerged in the network, with *Rule-Breaking Behavior* and *inhibitory control* as the primary parent nodes, indicating their critical importance in the DAG. *Rule-Breaking Behavior* and *inhibitory control* both have an in-degree of 0, meaning they have no incoming edges, but they exert significant influence with out-degrees of 8 and 5, respectively. *Inhibitory control* directly connects to four other EFs, *cognitive flexibility*, *working memory*, *processing speed*, and *episodic memory*, and one syndrome scale, *Social Problems*. *Cognitive Flexibility*, in turn, influences three EFs, *working memory*, *processing speed*, and *episodic memory*, and one syndrome scale, *Attention Problems*. *Working memory* further connects to four syndrome scales of *Thought Problems*, *Attention Problems*, *Social Problems*, *Anxious/Depressed* and two EFs, *episodic memory* and *processing speed*. *Episodic memory* influences *Anxious/Depressed* and *Attention Problems*, while *processing speed* connects to *Attention Problems*. *Attention Problems*, with seven incoming edges, and *working memory* with multiple outgoing connections may serve as key convergent nodes in the network.

The DAG in S2 Fig, derived from averaging 10,000 bootstrapped networks, illustrates the relationships between EFs and CBCL syndrome scales, with edge thickness reflecting the Bayesian Information Criterion (BIC) values from S2 Table. The highest BIC in the network was between *Rule-Breaking Behavior* and *Aggressive Behavior* (BIC = −2496.3), followed by *inhibitory control* and *cognitive flexibility* (BIC = −907.06). The thicker the edge, the more significant it is to model fit.

### Comparison of male and female sample networks

The male and female sample networks are presented in S3 Fig and S4 Fig, respectively, with their corresponding regularized partial correlation matrices provided in S3 Table and S4 Table, respectively. The NCT was conducted to compare these networks across three metrics: the Global Strength Invariance Test, Expected Influence (EI) centrality, and the Network Invariance Test. The results revealed no significant difference in EI centrality between the two networks, nor in the Global Strength Invariance Test. The global strengths were 5.08 for the male sample network and 5.27 for the female sample network, with a test statistic 𝑆 = 0.18 and a *p*-value of *0.3*, indicating no significant difference in global strength (*p > 0.05*). The Network Invariance Test, which evaluates whether the pattern of connections (edges) differs between the two networks, yielded a test statistic 𝑀 = 0.083 with a *p*-value of < *0.001*, indicating a significant difference in network structure between the male and female sample networks (*p < 0.05*). Subsequently, the Edge Invariance Test was performed to identify specific connections (edges) that differed between the male and female networks, with *p*-values adjusted using the Benjamini-Hochberg (BH) method to control for multiple comparisons. Significant differences (*p < 0.05*) were observed for two specific edges: *Social Problems* to *Aggressive Behavior* (*p* = *0.039*, test statistic 𝐸 = 0.07) and *Rule-Breaking Behavior* to *Aggressive Behavior* (*p* = *0.039*, test statistic 𝐸 = 0.086), indicating that these edges differ between males and females. The female network had greater edge strength from *Social Problems* to *Aggressive Behavior* (0.19) compared to the male network (0.13), indicating a stronger positive connection in females. Conversely, the male network displayed greater edge strength from *Rule-Breaking Behavior* to *Aggressive Behavior* (0.42) compared to the female network (0.33), reflecting a stronger positive connection in males. The results of the Edge Invariance Test are presented in S5 Table.

## Discussion

The study examined the associations and directional probabilities among EFs and dimensional psychopathology. We found modest associations among EFs and psychopathology, with *working memory* and *Attention Problems* emerging as central nodes within the network. *Inhibitory control* and externalizing psychopathology, specifically *Rule-Breaking Behavior*, emerged as parent nodes, underscoring their critical significance within the DAG. Significant sex differences were also observed. In the female network, the edge strength from *Social Problems* to A*ggressive Behavior* was stronger, whereas in the male network, the edge strength from *Rule-Breaking Behavior* to *Aggressive Behavior* was significantly greater than in the female network.

In the regularized partial correlation network, associations within clusters among EFs and among CBCL syndrome scales were generally stronger than associations between clusters. *Working memory* emerged as a central node in the network, exhibiting several connections to psychopathology. Better *working memory* performance was associated with fewer externalizing behaviors, including lower *Social Problems*, *Attention Problems*, and *Rule-Breaking Behavior*, which aligns with previous research linking *working memory* to externalizing psychopathology [81]**.** This finding is consistent with the theoretical accounts suggesting that *working memory* supports self-regulation and behavioral control [82]. Better *working memory* performance may help regulate attention, inhibit impulsive responses, and maintain goal-directed behavior, thereby mitigating externalizing behaviors [83,84].

Interestingly, *working memory* also exhibited inverse associations with internalizing behaviors, including *Anxious/Depressed*, *Thought Problems*, and *Somatic Complaints*. Although models of anxiety predict that poorer *working memory* should result from chronic rumination and worry [85,86], such findings are rarely observed empirically [87]. One plausible explanation is compensatory cognitive engagement, in which individuals experiencing mild internalizing symptoms may recruit *working memory* more intensively to manage worry or ruminative thoughts, effectively maintaining task performance despite internal distress [86,88,89]. This mechanism may account for the inverse associations observed in the network, suggesting that these links reflect adaptive engagement rather than deficits in cognitive functioning.

In DAG *inhibitory control* and externalizing behavior, specifically *Rule-Breaking Behavior*, emerged as primary drivers in the network, indicating their potential influence on both cognitive functions and psychopathology. *Inhibitory control* is a core EF that helps an individual inhibit a prepotent motor response, avoid distractions from irrelevant stimuli, and maintain a goal-directed behavior [21]. The direct edges of *inhibitory control* to other EFs may suggest a hierarchical organization, where it facilitates manipulation, updating and retrieval of information across these cognitive domains [21]. *Rule-breaking Behavior*, on the other hand, had several edges connecting to both the psychopathology and EFs, indicating that behavioral disinhibition may lead to broad range of consequences [90]**.**

Furthermore, the DAG in the current study revealed that externalizing problems, specifically *Rule-Breaking Behavior* and *Aggressive Behavior*, precede internalizing behaviors, including *Anxious/Depressed*, *Withdrawn/Depressed*, and *Somatic Complaints*. These findings support the developmental cascade model, which posits that externalizing problems often precipitate internalizing symptoms, rather than the reverse [90,91]. Externalizing behaviors, such as aggression or rule-breaking, may elicit adverse environmental consequences, including peer rejection, academic difficulties, or punitive responses. These negative experiences in turn can contribute to the development and emergence of internalizing symptoms.

*Working memory* and *Attention Problems* emerged as convergent nodes, exerting broad influence across the network in the DAG. The directed edges from *working memory* to several syndrome scales, including *Attention Problems*, *Thought Problems*, *Anxious/Depressed*, and *Social Problems*, suggest that *working memory* may serve as a core cognitive mechanism underlying a range of dimensional psychopathologies. This interpretation aligns with meta-analytic evidence showing that *working memory* deficits contribute to emotional vulnerability, attentional dysregulation, and impairments in social functioning [92,93]. The directed edges from *working memory* to other EFs in the DAG further indicate that *working memory* may operate as a cognitive hub, integrates information across cognitive domains to support adaptive functioning [94].

*Attention Problems*, on the other hand, received the highest number of incoming directed edges, suggesting that it may function as a key outcome node influenced by multiple upstream processes. This pattern is consistent with cognitive control theories, which conceptualize attentional dysregulation as a common endpoint of executive dysfunction and emotional interference [85,86]. Together, these findings imply that *inhibitory control* and *working memory* may jointly influence attentional regulation, thereby increasing vulnerability to both internalizing and externalizing behaviors.

The findings of the present study can be meaningfully interpreted within the RDoC framework, which emphasizes dimensional constructs that transcend traditional categorical diagnoses (17). *Working memory*, identified as a central node, aligns with the Cognitive Systems domain, reflecting its critical role in goal-directed behavior and attentional regulation. The observed associations between EFs and both internalizing and externalizing psychopathology map onto the Negative and Positive Valence Systems, as well as the Social Processes domain (17). These patterns likely reflect cross-domain interactions supporting emotional regulation, social functioning, and behavioral control, signifying the transdiagnostic role of EFs across dimensions of psychopathology.

The edge weight between *Social Problems* and *Aggressive Behavior* was stronger in females than in males, while the edge weight between *Rule-Breaking Behavior* and *Aggressive Behavior* was stronger in males compared to females in GGM networks. The stronger association between *Social Problems* and *Aggressive Behavior* in females may reflect how social difficulties are more intimately tied to emotional dysregulation and aggression in girls. Females tend to place a higher emotional value on peer relationships and social inclusion during preadolescence [95]. Disruptions in social functioning such as peer rejection, exclusion, or interpersonal conflict may elicit heightened emotional distress, which can manifest as reactive or relational aggression [96]. This finding aligns with relational aggression theory, which posits that girls are more likely to express aggression through social manipulation or exclusion rather than overt physical acts [97]. Consequently, when girls experience social problems, they may be more likely to respond with aggressive behaviors rooted in interpersonal distress. In contrast, the stronger association between *Rule-Breaking Behavior* and *Aggressive Behavior* in males may reflect boys’ greater tendency to engage in disruptive, overt, and externalizing behaviors. On average, males exhibit higher levels of impulsivity and sensation-seeking, and are more likely to externalize distress or frustration through physical aggression or defiance [98,99]. These behaviors are often conceptualized within a behavioral cascade model, where early rule-breaking behaviors (e.g., defiance, lying) can escalate into more severe antisocial conduct, such as aggression [100].

### Implications and Limitations

The present study offers valuable insights into the relationship between EFs and dimensional psychopathology in preadolescents. By examining associations between multiple EFs and syndrome scales derived from the CBCL, the findings contribute to a growing body of research supporting the transdiagnostic relevance of cognitive processes in mental health. *Working memory* and *inhibitory control*, as central and precursor nodes respectively, may jointly shape both cognitive and emotional development. Among the EFs, *working memory* emerged as particularly significant, demonstrating consistent associations with a range of both internalizing and externalizing problems. This suggests that *working memory* may serve as a transdiagnostic cognitive mechanism implicated across multiple forms of psychopathology. *Cognitive flexibility* and *inhibitory control* were also linked to several syndrome scales, highlighting their broader roles in emotional and behavioral regulation. Cognitive training interventions targeting *working memory* and *inhibitory control* may have broad transdiagnostic benefits by enhancing self-regulation and attentional control, thereby reducing vulnerability to both internalizing and externalizing behaviors. These findings signify the complex interplay among different EF components and diverse symptom dimensions, emphasizing the potential value of targeted interventions aimed at specific EF deficits to improve mental health outcomes in youth.

Several limitations of the present study should be acknowledged. First, the cross-sectional design limits the ability to draw causal inferences, despite the use of DAG to explore potential directional relationships. Second, dimensional psychopathology was based on parent-reported CBCL, which may introduce bias due to parental perceptions. Third, the sample consisted of non-clinical preadolescents, which may restrict the generalizability of the findings to clinical populations or other age groups. Future research should aim to replicate these findings in transdiagnostic clinical samples and across a broader developmental range. Fourth, the ABCD study sample has been criticized as being self-selected and may not be fully representative of the U.S. population [101]. Lastly, this study was based on a single large sample without an independent replication dataset, which may limit the generalizability of the findings. Future research should aim to replicate and validate these results using independent samples, or machine learning approaches to further evaluate the stability and generalizability of the findings.

## Conclusions

The findings revealed modest but robust associations between EFs and dimensional psychopathology, supported by regularized partial correlations in a large, well-powered sample. *Working memory* emerged as a central transdiagnostic hub, exhibiting associations across multiple behavioral domains, with *Attention Problems* also identified as a key hub in both the partial correlation network and DAG analyses. The DAG further suggested that externalizing problems may cascade into internalizing problems, underscoring potential developmental pathways. Sex-stratified analyses revealed nuanced differences, emphasizing the importance of considering sex in developmental psychopathology research. Collectively, these results advance our understanding of the cognitive underpinnings of dimensional psychopathology, support dimensional models such as RDoC [17], and provide a foundation for future research aimed at targeted interventions and preventive strategies in adolescents.

## Supporting information

S1 Table Regularized partial correlation of the EFs and CBCL syndrome scales. (DOCX)

S2 Table BIC values of arrows in the DAG. (DOCX)

S3 Table Regularized partial correlation matrix of male sample. (DOCX)

S4 Table Regularized partial correlation matrix of female sample. (DOCX)

S5 Table Edge Invariance Test. (DOCX)

S1 Fig Bootstrap edge stability graph of the EF-psychopathology network. (TIF)

S2 Fig Directed Acyclic Graph of EFs and CBCL syndrome scales with arrow thickness indicating the importance of each arrow to the overall network model fit. (TIF)

S3 Fig Male sample EF-psychopathology network. (TIF)

S4 Fig Female sample EF-psychopathology network. (TIF)

S1 File Study Measures. (DOCX)
