# Supplementary material for: Executive functions and psychopathology: A transdiagnostic network analysis
Source: PLoS One. 2025 Dec 26;20(12):e0338435. doi: 10.1371/journal.pone.0338435 (PMC12742799; doi:10.1371/journal.pone.0338435)
Supplement: S1 Table — (DOCX) [file pone.0338435.s001.docx]

**S1 Table. Regularized partial correlation of the EFs and CBCL syndrome scales.**

|  | AnxDep | WithDep | SomCom | SocProb | ThoProb | AttProb | RuBBeh | AggBeh | InhCon | WorkMem | CogFlex | ProcSp | EpMem |
| --- | --- | --- | --- | --- | --- | --- | --- | --- | --- | --- | --- | --- | --- |
| AnxDep | 0.00 | 0.28 | 0.17 | 0.21 | 0.19 | 0.01 | -0.04 | 0.12 | 0.00 | -0.04 | -0.01 | 0.00 | -0.03 |
| WithDep | 0.28 | 0.00 | 0.07 | 0.16 | 0.08 | 0.05 | 0.05 | 0.04 | 0.00 | 0.00 | 0.02 | -0.02 | 0.00 |
| SomCom | 0.17 | 0.07 | 0.00 | 0.07 | 0.13 | 0.00 | 0.01 | 0.07 | 0.00 | -0.01 | 0.00 | -0.02 | 0.00 |
| SocProb | 0.21 | 0.16 | 0.07 | 0.00 | 0.08 | 0.21 | 0.12 | 0.15 | 0.02 | 0.06 | 0.01 | 0.02 | 0.02 |
| ThoProb | 0.19 | 0.08 | 0.13 | 0.08 | 0.00 | 0.28 | 0.06 | 0.09 | 0.00 | -0.03 | -0.01 | -0.01 | -0.01 |
| AttProb | 0.01 | 0.05 | 0.00 | 0.21 | 0.28 | 0.00 | 0.14 | 0.21 | 0.02 | 0.03 | 0.02 | 0.05 | 0.04 |
| RuBBeh | -0.04 | 0.05 | 0.01 | 0.12 | 0.06 | 0.14 | 0.00 | 0.40 | -0.02 | 0.02 | 0.01 | -0.01 | 0.04 |
| AggBeh | 0.12 | 0.04 | 0.07 | 0.15 | 0.09 | 0.21 | 0.40 | 0.00 | 0.00 | 0.00 | -0.01 | 0.00 | 0.00 |
| InhCon | 0.00 | 0.00 | 0.00 | 0.02 | 0.00 | 0.02 | -0.02 | 0.00 | 0.00 | 0.12 | 0.30 | 0.20 | 0.03 |
| WorkMem | -0.04 | 0.00 | -0.01 | 0.06 | -0.03 | 0.03 | 0.02 | 0.00 | 0.12 | 0.00 | 0.10 | 0.03 | 0.26 |
| CogFlex | -0.01 | 0.02 | 0.00 | 0.01 | -0.01 | 0.02 | 0.01 | -0.01 | 0.30 | 0.10 | 0.00 | 0.28 | 0.10 |
| ProcSp | 0.00 | -0.02 | -0.02 | 0.02 | -0.01 | 0.05 | -0.01 | 0.00 | 0.20 | 0.03 | 0.28 | 0.00 | 0.05 |
| EpMem | -0.03 | 0.00 | 0.00 | 0.02 | -0.01 | 0.04 | 0.04 | 0.00 | 0.03 | 0.26 | 0.10 | 0.05 | 0.00 |

***Notes:*** AnxDep is Anxious/Depressed; WithDep isWithdrawn/Depressed; SomComp is Somatic Complaints; SocProb is Social Problems; ThouProb is Thought Problems; AttProb is Attention Problems; RuBBeh is Rule-Breaking Behavior; AggBeh is Aggressive Behavior; InhCon is Inhibitory Control; WorkMem is Working Memory; CogFlex is Cognitive Flexibility; ProcSp is Processing Speed; and EpMem is Episodic Memory.
