# Supplementary material for: Executive functions and psychopathology: A transdiagnostic network analysis
Source: PLoS One. 2025 Dec 26;20(12):e0338435. doi: 10.1371/journal.pone.0338435 (PMC12742799; doi:10.1371/journal.pone.0338435)
Supplement: S2 Table — (DOCX) [file pone.0338435.s002.docx]

**S2 Table. BIC values of arrows in the DAG.**

| **Arrows in the DAG** | | **Values determining arrow thickness** |
| --- | --- | --- |
| **From** | **To** | **BIC** |
| AnxDep | WithDep | -425.99 |
| AnxDep | SomCom | -125.73 |
| AnxDep | ThoProb | -298.82 |
| WithDep | SomCom | -17.44 |
| SocProb | AnxDep | -710.37 |
| SocProb | WithDep | -125.21 |
| SocProb | SomCom | -21.25 |
| SocProb | ThoProb | -42.56 |
| SocProb | AttProb | -476.74 |
| ThoProb | WithDep | -28.73 |
| ThoProb | SomCom | -85.08 |
| AttProb | WithDep | -5.027 |
| AttProb | ThoProb | -383.48 |
| RuBBeh | WithDep | -11.49 |
| RuBBeh | SocProb | -208.77 |
| RuBBeh | ThoProb | -22.60 |
| RuBBeh | AttProb | -123.14 |
| RuBBeh | AggBeh | -2496.3 |
| RuBBeh | WorkMem | -26.26 |
| RuBBeh | CogFlex | -18.60 |
| RuBBeh | EpMem | -21.57 |
| AggBeh | AnxDep | -325.17 |
| AggBeh | WithDep | -4.913 |
| AggBeh | SomCom | -25.91 |
| AggBeh | SocProb | -748.57 |
| AggBeh | ThoProb | -42.94 |
| AggBeh | AttProb | -381.65 |
| InhCon | SocProb | -9.31 |
| InhCon | WorkMem | -97.49 |
| InhCon | CogFlex | -907.06 |
| InhCon | ProcSp | -189.09 |
| InhCon | EpMem | -4.21 |
| WorkMem | AnxDep | -16.99 |
| WorkMem | SocProb | -18.61 |
| WorkMem | ThoProb | -11.63 |
| WorkMem | AttProb | 2.89 |
| WorkMem | ProcSp | -0.41 |
| WorkMem | EpMem | -337.24 |
| CogFlex | AttProb | 0.38 |
| CogFlex | WorkMem | -117.57 |
| CogFlex | ProcSp | -382.63 |
| CogFlex | EpMem | -69.49 |
| ProcSp | AttProb | -11.88 |
| EpMem | AnxDep | -1.47 |
| EpMem | AttProb | -2.11 |
| EpMem | ProcSp | -8.33 |

***Notes:*** AnxDep is Anxious/Depressed; WithDep isWithdrawn/Depressed; SomComp is Somatic Complaints; SocProb is Social Problems; ThouProb is Thought Problems; AttProb is Attention Problems; RuBBeh is Rule-Breaking Behavior; AggBeh is Aggressive Behavior; InhCon is Inhibitory Control; WorkMem is Working Memory; CogFlex is Cognitive Flexibility; ProcSp is Processing Speed; and EpMem is Episodic Memory.
