# Supplementary material for: Executive functions and psychopathology: A transdiagnostic network analysis
Source: PLoS One. 2025 Dec 26;20(12):e0338435. doi: 10.1371/journal.pone.0338435 (PMC12742799; doi:10.1371/journal.pone.0338435)
Supplement: S3 Table — (DOCX) [file pone.0338435.s003.docx]

**S3 Table. Regularized partial correlation matrix of male sample.**

|  | AnxDep | WithDep | SomCom | SocProb | ThoProb | AttProb | RuBBeh | AggBeh | InhCon | WorkMem | CogFlex | ProcSp | EpMem |
| --- | --- | --- | --- | --- | --- | --- | --- | --- | --- | --- | --- | --- | --- |
| AnxDep | 0.0000 | 0.2806 | 0.1269 | 0.1693 | 0.1983 | 0.0008 | 0.0000 | 0.1200 | 0.0000 | -0.0202 | 0.0000 | 0.0000 | -0.0050 |
| WithDep | 0.2806 | 0.0000 | 0.0750 | 0.1754 | 0.0820 | 0.0456 | 0.0252 | 0.0433 | 0.0000 | 0.0000 | 0.0000 | 0.0000 | 0.0000 |
| SomCom | 0.1269 | 0.0750 | 0.0000 | 0.0801 | 0.1300 | 0.0000 | 0.0072 | 0.0740 | 0.0000 | 0.0000 | 0.0000 | -0.0103 | 0.0000 |
| SocProb | 0.1693 | 0.1754 | 0.0801 | 0.0000 | 0.1031 | 0.2491 | 0.0982 | 0.1226 | 0.0160 | 0.0518 | 0.0000 | 0.0117 | 0.0057 |
| ThoProb | 0.1983 | 0.0820 | 0.1300 | 0.1031 | 0.0000 | 0.2648 | 0.0500 | 0.0857 | -0.0038 | -0.0251 | 0.0000 | 0.0000 | 0.0000 |
| AttProb | 0.0008 | 0.0456 | 0.0000 | 0.2491 | 0.2648 | 0.0000 | 0.1269 | 0.2176 | 0.0204 | 0.0262 | 0.0126 | 0.0298 | 0.0242 |
| RuBBeh | 0.0000 | 0.0252 | 0.0072 | 0.0982 | 0.0500 | 0.1269 | 0.0000 | 0.4188 | 0.0000 | 0.0195 | 0.0000 | 0.0000 | 0.0114 |
| AggBeh | 0.1200 | 0.0433 | 0.0740 | 0.1226 | 0.0857 | 0.2176 | 0.4188 | 0.0000 | 0.0000 | 0.0000 | 0.0000 | 0.0000 | 0.0000 |
| InhCon | 0.0000 | 0.0000 | 0.0000 | 0.0160 | -0.0038 | 0.0204 | 0.0000 | 0.0000 | 0.0000 | 0.1066 | 0.2697 | 0.1947 | 0.0295 |
| WorkMem | -0.0202 | 0.0000 | 0.0000 | 0.0518 | -0.0251 | 0.0262 | 0.0195 | 0.0000 | 0.1066 | 0.0000 | 0.1175 | 0.0361 | 0.2449 |
| CogFlex | 0.0000 | 0.0000 | 0.0000 | 0.0000 | 0.0000 | 0.0126 | 0.0000 | 0.0000 | 0.2697 | 0.1175 | 0.0000 | 0.2761 | 0.1180 |
| ProcSp | 0.0000 | 0.0000 | -0.0103 | 0.0117 | 0.0000 | 0.0298 | 0.0000 | 0.0000 | 0.1947 | 0.0361 | 0.2761 | 0.0000 | 0.0144 |
| EpMem | -0.0050 | 0.0000 | 0.0000 | 0.0057 | 0.0000 | 0.0242 | 0.0114 | 0.0000 | 0.0295 | 0.2449 | 0.1180 | 0.0144 | 0.0000 |

***Notes:*** AnxDep is Anxious/Depressed; WithDep isWithdrawn/Depressed; SomComp is Somatic Complaints; SocProb is Social Problems; ThouProb is Thought Problems; AttProb is Attention Problems; RuBBeh is Rule-Breaking Behavior; AggBeh is Aggressive Behavior; InhCon is Inhibitory Control; WorkMem is Working Memory; CogFlex is Cognitive Flexibility; ProcSp is Processing Speed; and EpMem is Episodic Memory.
