# Supplementary material for: Executive functions and psychopathology: A transdiagnostic network analysis
Source: PLoS One. 2025 Dec 26;20(12):e0338435. doi: 10.1371/journal.pone.0338435 (PMC12742799; doi:10.1371/journal.pone.0338435)
Supplement: S4 Table — (DOCX) [file pone.0338435.s004.docx]

**S4 Table. Regularized partial correlation matrix of female sample.**

|  | AnxDep | WithDep | SomCom | SocProb | ThoProb | AttProb | RuBBeh | AggBeh | InhCon | WorkMem | CogFlex | ProcSp | EpMem |
| --- | --- | --- | --- | --- | --- | --- | --- | --- | --- | --- | --- | --- | --- |
| AnxDep | 0.0000 | 0.2684 | 0.1877 | 0.2182 | 0.1765 | 0.0222 | 0.0000 | 0.0970 | 0.0000 | -0.0499 | 0.0000 | 0.0000 | -0.0082 |
| WithDep | 0.2684 | 0.0000 | 0.0664 | 0.1456 | 0.0637 | 0.0432 | 0.0543 | 0.0598 | 0.0000 | 0.0000 | 0.0000 | -0.0053 | 0.0000 |
| SomCom | 0.1877 | 0.0664 | 0.0000 | 0.0556 | 0.1438 | 0.0098 | 0.0079 | 0.0723 | 0.0000 | -0.0143 | 0.0000 | 0.0000 | 0.0000 |
| SocProb | 0.2182 | 0.1456 | 0.0556 | 0.0000 | 0.0484 | 0.1852 | 0.1501 | 0.1930 | 0.0046 | 0.0453 | 0.0169 | 0.0036 | 0.0147 |
| ThoProb | 0.1765 | 0.0637 | 0.1438 | 0.0484 | 0.0000 | 0.2522 | 0.0560 | 0.1065 | 0.0000 | -0.0021 | 0.0000 | 0.0000 | 0.0000 |
| AttProb | 0.0222 | 0.0432 | 0.0098 | 0.1852 | 0.2522 | 0.0000 | 0.1293 | 0.1953 | 0.0153 | 0.0327 | 0.0192 | 0.0336 | 0.0239 |
| RuBBeh | 0.0000 | 0.0543 | 0.0079 | 0.1501 | 0.0560 | 0.1293 | 0.0000 | 0.3333 | 0.0000 | 0.0197 | 0.0062 | 0.0000 | 0.0391 |
| AggBeh | 0.0970 | 0.0598 | 0.0723 | 0.1930 | 0.1065 | 0.1953 | 0.3333 | 0.0000 | 0.0000 | 0.0000 | 0.0000 | 0.0000 | 0.0000 |
| InhCon | 0.0000 | 0.0000 | 0.0000 | 0.0046 | 0.0000 | 0.0153 | 0.0000 | 0.0000 | 0.0000 | 0.1109 | 0.3183 | 0.2014 | 0.0318 |
| WorkMem | -0.0499 | 0.0000 | -0.0143 | 0.0453 | -0.0021 | 0.0327 | 0.0197 | 0.0000 | 0.1109 | 0.0000 | 0.0898 | 0.0202 | 0.2671 |
| CogFlex | 0.0000 | 0.0000 | 0.0000 | 0.0169 | 0.0000 | 0.0192 | 0.0062 | 0.0000 | 0.3183 | 0.0898 | 0.0000 | 0.2654 | 0.0792 |
| ProcSp | 0.0000 | -0.0053 | 0.0000 | 0.0036 | 0.0000 | 0.0336 | 0.0000 | 0.0000 | 0.2014 | 0.0202 | 0.2654 | 0.0000 | 0.0674 |
| EpMem | -0.0082 | 0.0000 | 0.0000 | 0.0147 | 0.0000 | 0.0239 | 0.0391 | 0.0000 | 0.0318 | 0.2671 | 0.0792 | 0.0674 | 0.0000 |

***Notes:*** AnxDep is Anxious/Depressed; WithDep isWithdrawn/Depressed; SomComp is Somatic Complaints; SocProb is Social Problems; ThouProb is Thought Problems; AttProb is Attention Problems; RuBBeh is Rule-Breaking Behavior; AggBeh is Aggressive Behavior; InhCon is Inhibitory Control; WorkMem is Working Memory; CogFlex is Cognitive Flexibility; ProcSp is Processing Speed; and EpMem is Episodic Memory.
