# Supplementary material for: Executive functions and psychopathology: A transdiagnostic network analysis
Source: PLoS One. 2025 Dec 26;20(12):e0338435. doi: 10.1371/journal.pone.0338435 (PMC12742799; doi:10.1371/journal.pone.0338435)
Supplement: S5 Table — (DOCX) [file pone.0338435.s005.docx]

**S5 Table. Edge Invariance Test.**

| **Var1** | **Var2** | ***p*-value** | **Test statistic E** |
| --- | --- | --- | --- |
| AnxDep | WithDep | 1.000 | 0.012 |
| AnxDep | SomCom | 0.097 | 0.061 |
| WithDep | SomCom | 1.000 | 0.009 |
| AnxDep | SocProb | 0.190 | 0.049 |
| WithDep | SocProb | 0.831 | 0.030 |
| SomCom | SocProb | 0.885 | 0.024 |
| AnxDep | ThoProb | 0.885 | 0.022 |
| WithDep | ThoProb | 1.000 | 0.018 |
| SomCom | ThoProb | 1.000 | 0.014 |
| SocProb | ThoProb | 0.109 | 0.055 |
| AnxDep | AttProb | 0.327 | 0.021 |
| WithDep | AttProb | 1.000 | 0.002 |
| SomCom | AttProb | 0.519 | 0.010 |
| SocProb | AttProb | 0.078 | 0.064 |
| ThoProb | AttProb | 1.000 | 0.013 |
| AnxDep | RuBBeh | 1.000 | 0.000 |
| WithDep | RuBBeh | 0.833 | 0.029 |
| SomCom | RuBBeh | 1.000 | 0.001 |
| SocProb | RuBBeh | 0.190 | 0.052 |
| ThoProb | RuBBeh | 1.000 | 0.006 |
| AttProb | RuBBeh | 1.000 | 0.002 |
| AnxDep | AggBeh | 0.885 | 0.023 |
| WithDep | AggBeh | 1.000 | 0.016 |
| SomCom | AggBeh | 1.000 | 0.002 |
| SocProb | AggBeh | 0.039 | 0.070 |
| ThoProb | AggBeh | 0.938 | 0.021 |
| AttProb | AggBeh | 0.885 | 0.022 |
| RuBBeh | AggBeh | 0.039 | 0.086 |
| AnxDep | InhCon | 1.000 | 0.000 |
| WithDep | InhCon | 1.000 | 0.000 |
| SomCom | InhCon | 1.000 | 0.000 |
| SocProb | InhCon | 1.000 | 0.011 |
| ThoProb | InhCon | 0.833 | 0.004 |
| AttProb | InhCon | 1.000 | 0.005 |
| RuBBeh | InhCon | 1.000 | 0.000 |
| AggBeh | InhCon | 1.000 | 0.000 |
| AnxDep | WorkMem | 0.533 | 0.030 |
| WithDep | WorkMem | 1.000 | 0.000 |
| SomCom | WorkMem | 0.938 | 0.014 |
| SocProb | WorkMem | 1.000 | 0.006 |
| ThoProb | WorkMem | 0.885 | 0.023 |
| AttProb | WorkMem | 1.000 | 0.006 |
| RuBBeh | WorkMem | 1.000 | 0.000 |
| AggBeh | WorkMem | 1.000 | 0.000 |
| InhCon | WorkMem | 1.000 | 0.004 |
| AnxDep | CogFlex | 1.000 | 0.000 |
| WithDep | CogFlex | 1.000 | 0.000 |
| SomCom | CogFlex | 1.000 | 0.000 |
| SocProb | CogFlex | 0.833 | 0.017 |
| ThoProb | CogFlex | 1.000 | 0.000 |
| AttProb | CogFlex | 1.000 | 0.007 |
| RuBBeh | CogFlex | 1.000 | 0.006 |
| AggBeh | CogFlex | 1.000 | 0.000 |
| InhCon | CogFlex | 0.156 | 0.049 |
| WorkMem | CogFlex | 0.833 | 0.028 |
| AnxDep | ProcSp | 1.000 | 0.000 |
| WithDep | ProcSp | 1.000 | 0.005 |
| SomCom | ProcSp | 1.000 | 0.010 |
| SocProb | ProcSp | 1.000 | 0.008 |
| ThoProb | ProcSp | 1.000 | 0.000 |
| AttProb | ProcSp | 1.000 | 0.004 |
| RuBBeh | ProcSp | 1.000 | 0.000 |
| AggBeh | ProcSp | 1.000 | 0.000 |
| InhCon | ProcSp | 1.000 | 0.007 |
| WorkMem | ProcSp | 1.000 | 0.016 |
| CogFlex | ProcSp | 1.000 | 0.011 |
| AnxDep | EpMem | 1.000 | 0.003 |
| WithDep | EpMem | 1.000 | 0.000 |
| SomCom | EpMem | 1.000 | 0.000 |
| SocProb | EpMem | 1.000 | 0.009 |
| ThoProb | EpMem | 1.000 | 0.000 |
| AttProb | EpMem | 1.000 | 0.000 |
| RuBBeh | EpMem | 0.740 | 0.028 |
| AggBeh | EpMem | 1.000 | 0.000 |
| InhCon | EpMem | 1.000 | 0.002 |
| WorkMem | EpMem | 0.885 | 0.022 |
| CogFlex | EpMem | 0.489 | 0.039 |
| ProcSp | EpMem | 0.143 | 0.053 |

***Notes:*** AnxDep is Anxious/Depressed; WithDep isWithdrawn/Depressed; SomComp is Somatic Complaints; SocProb is Social Problems; ThouProb is Thought Problems; AttProb is Attention Problems; RuBBeh is Rule-Breaking Behavior; AggBeh is Aggressive Behavior; InhCon is Inhibitory Control; WorkMem is Working Memory; CogFlex is Cognitive Flexibility; ProcSp is Processing Speed; and EpMem is Episodic Memory.
