# Supplementary figures and images for: Executive functions and psychopathology: A transdiagnostic network analysis
Source: PLoS One. 2025 Dec 26;20(12):e0338435. doi: 10.1371/journal.pone.0338435 (PMC12742799; doi:10.1371/journal.pone.0338435)

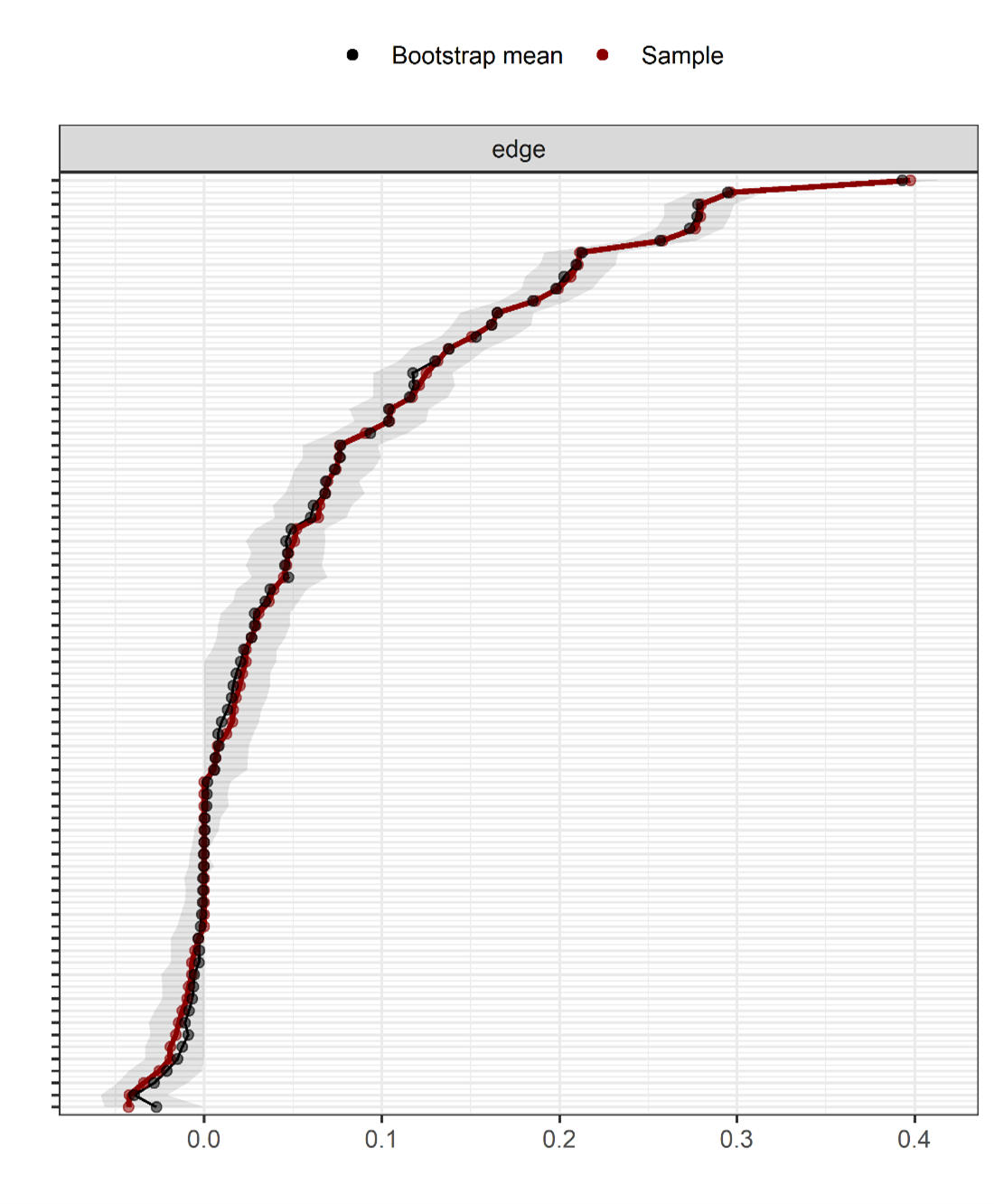

Supplement: S1 Fig — (TIF) [file pone.0338435.s006.tif]

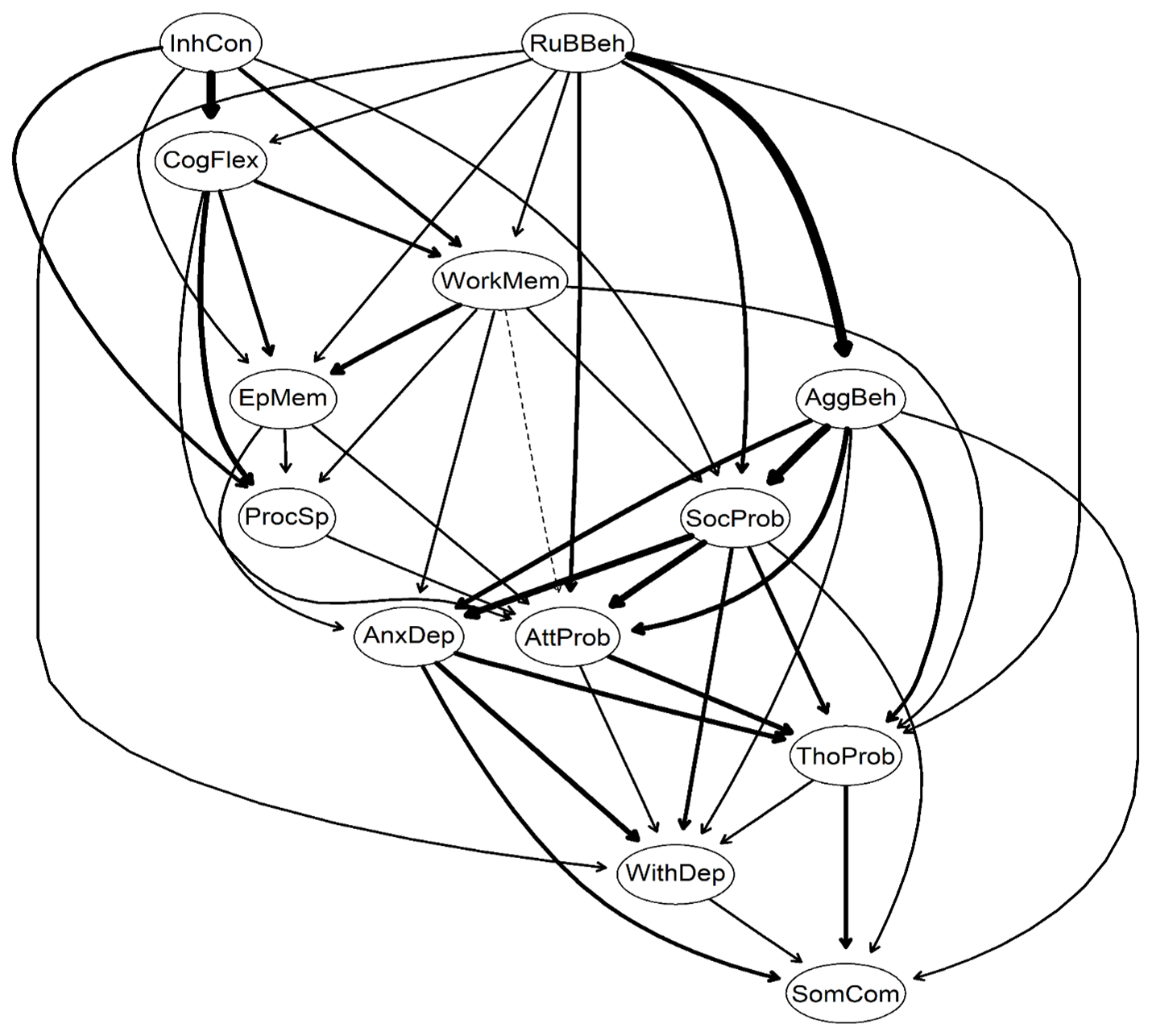

Supplement: S2 Fig — (TIF) [file pone.0338435.s007.tif]

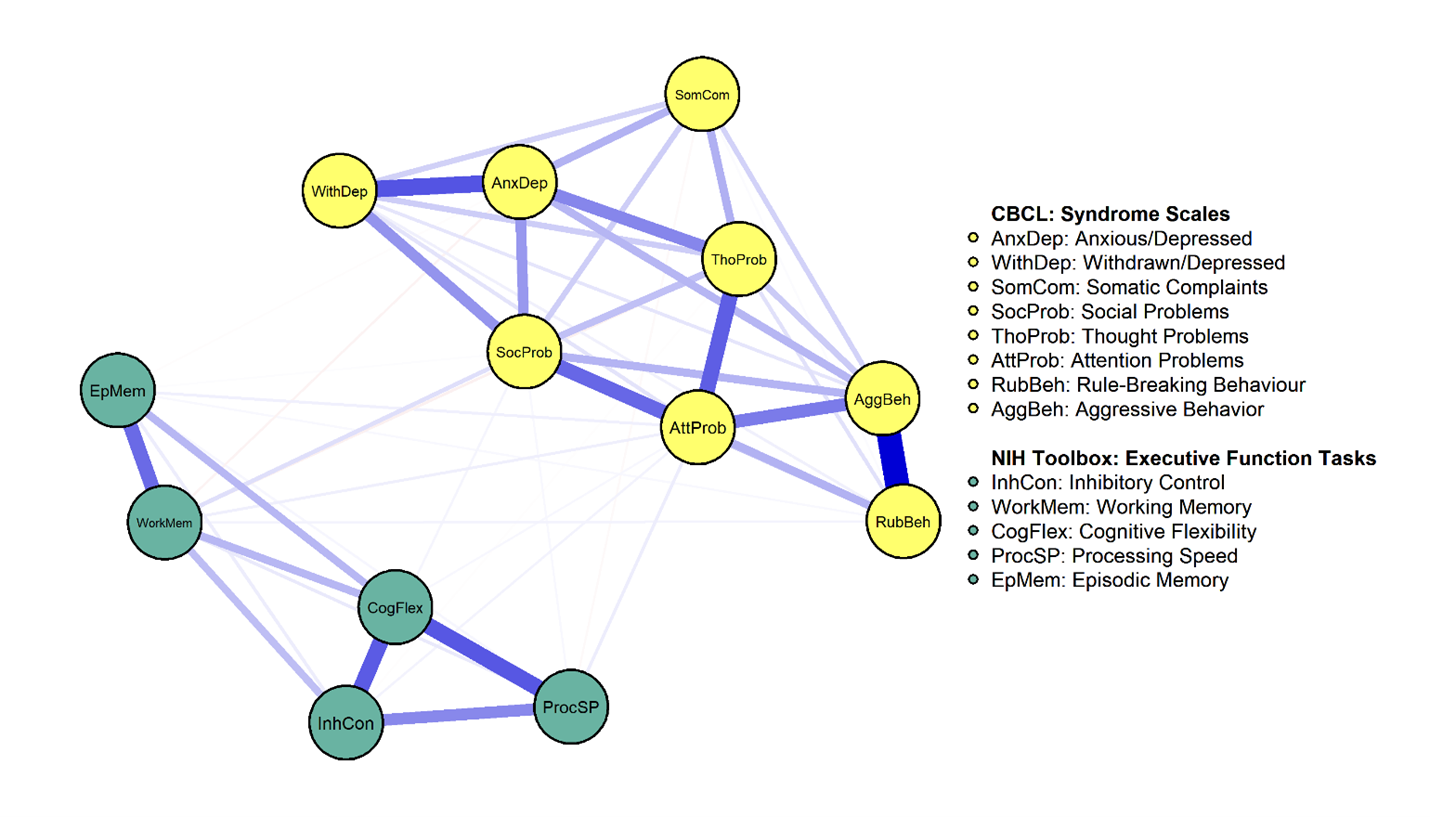

Supplement: S3 Fig — (TIF) [file pone.0338435.s008.tif]

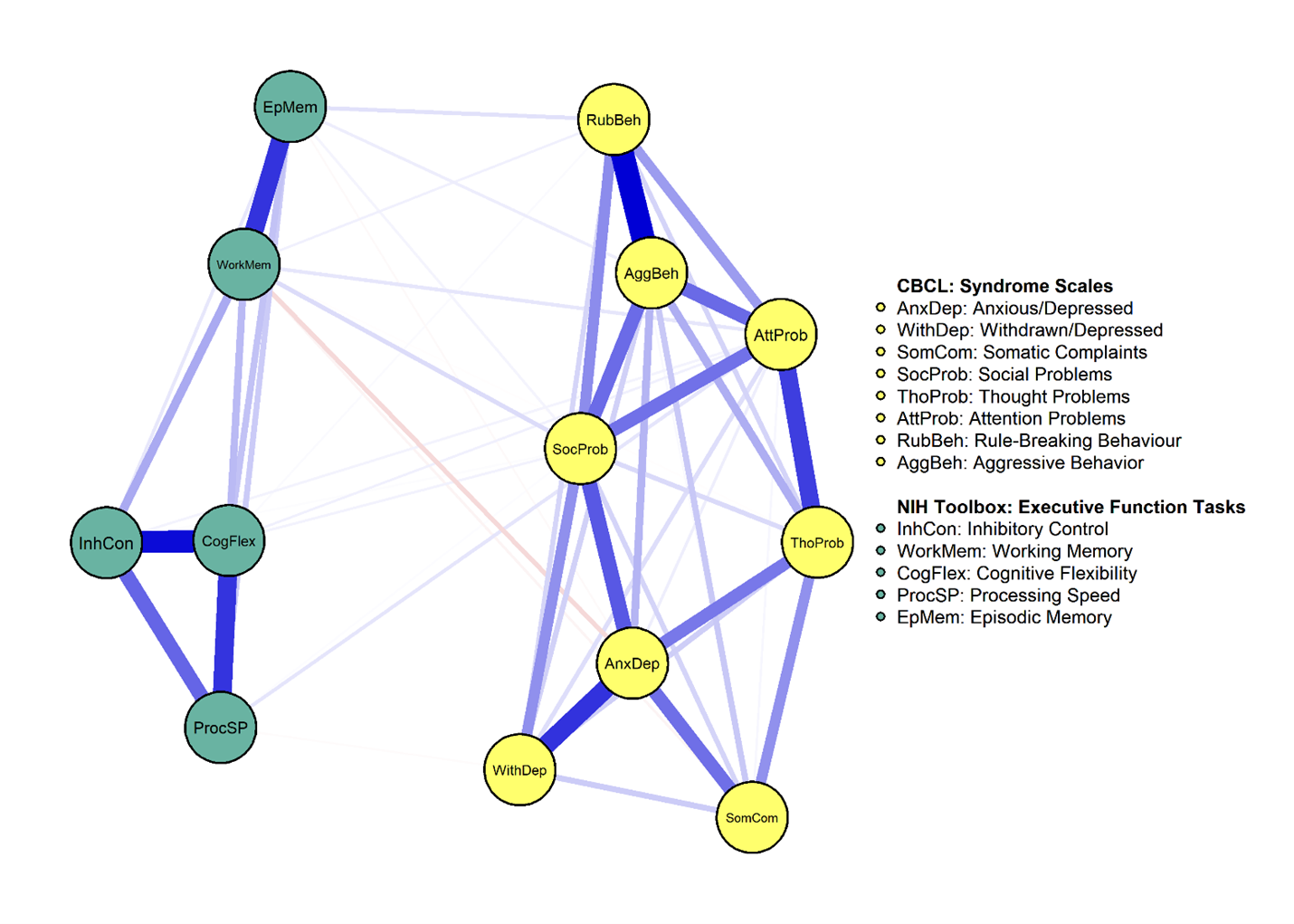

Supplement: S4 Fig — (TIF) [file pone.0338435.s009.tif]
