# Supplementary material for: Executive functions and psychopathology: A transdiagnostic network analysis
Source: PLoS One. 2025 Dec 26;20(12):e0338435. doi: 10.1371/journal.pone.0338435 (PMC12742799; doi:10.1371/journal.pone.0338435)
Supplement: S1 File — (DOCX) [file pone.0338435.s010.docx]

**Study Measures**

Participant demographics were obtained parent demographic survey (Pdem02), race/ethnicity from the ACS post stratification weights (acspsw03), and medical history from the parent medical history questionnaire (MHX; abcd_mx01).

Raw dimensional psychopathology scores were drawn from the CBCL (abcd_cbcls01): AnxDep (cbcl_scr_syn_anxdep_r), WithDep (cbcl_scr_syn_withdep_r), SomCom (cbcl_scr_syn_somatic_r), SocProb (cbcl_scr_syn_social_r), ThoProb (cbcl_scr_syn_thought_r), AttProb (cbcl_scr_syn_attention_r), RuBBeh (cbcl_scr_syn_rulebreak_r), and AggBeh (cbcl_scr_syn_aggressive_r).

Executive function measures were obtained from NIH Toolbox (abcd_tbss01): InhCon (nihtbx_flanker_agecorrected), WorkMem (nihtbx_list_agecorrected), CogFlex (nihtbx_cardsort_agecorrected), and EpMem (nihtbx_picture_agecorrected).
